# Systematic and Random Mapping Errors in Structure – Function Analysis of the Macula

**DOI:** 10.1167/tvst.10.2.21

**Published:** 2021-02-16

**Authors:** Giovanni Montesano, Luca M. Rossetti, Davide Allegrini, Mario R. Romano, David F. Garway-Heath, David P. Crabb

**Affiliations:** 1 Optometry and Visual Sciences, City, University of London, London, UK; 2 ASST Santi Paolo e Carlo, University of Milan, Milan, Italy; 3 NIHR Biomedical Research Centre, Moorfields Eye Hospital NHS Foundation Trust, UCL Institute of Ophthalmology, London, UK; 4 Eye Unit, Humanitas Gavazzeni Hospital, Humanitas University, Bergamo, Italy

**Keywords:** glaucoma, optical coherence tomography, visual field, perimetry, fixation

## Abstract

**Purpose:**

Quantify the spatial error in mapping perimetric stimuli for structure–function analysis resulting from the choice of mapping scheme and eye movements.

**Methods:**

We analyzed data from 17 healthy and 30 glaucomatous participants. Structural data of the macula were collected with a spectral-domain optical coherence tomography. We extracted eye movement data and projection locations from a fundus tracking perimeter and quantified the retinal location mapping error (distance between the actual and the intended stimulus location in degrees from the fovea) for non-tracked perimetry in a 10-2 grid. First, we evaluated whether rotating the 10-2 grid to match the fovea–disc axis improved mapping accuracy. Second, we analyzed the effect of eccentric fixation, random eye movements, and gaze attraction from seen stimuli on projection accuracy and spread of fixation, measured with the 95% bivariate contour ellipse area (95% BCEA). We used generalized linear mixed models for our statistical analyses.

**Results:**

Rotating the 10-2 grid to match the fovea–disc axis significantly increased the mapping error (*P* < 0.001). Eye movements evoked by seen stimuli significantly increased the projection error during the test (*P* < 0.001). Removing such eye movements significantly reduced the 95% BCEA (*P* < 0.001). Eccentric fixation also significantly contributed to the projection error (*P* < 0.001), and its effect was larger in glaucoma patients (*P* < 0.001).

**Conclusions:**

Rotating the perimetric grid to match the fovea–disc axis is not recommended. Fixation eccentricity and instability should be taken into account for structure–function analyses.

**Translational Relevance:**

Accounting for fixation can improve structure–function mapping in glaucoma.

## Introduction

Glaucoma is characterized by structural loss of neural tissue and associated functional damage to the visual field (VF). Therefore, spatial mapping of the location of visual function measurements to image-based measurements of retinal structure is important when evaluating the agreement of estimates of functional and structural damage.

Optical coherence tomography (OCT) is widely used to provide a quantitative three-dimensional assessment of thickness of different layers of the retina and optic nerve head (ONH).^1^^,^^2^ The most affected layers in glaucoma are the retinal nerve fiber layer and the ganglion cell layer, which typically show localized or diffused thinning when damaged.^1^^–^^3^ Functional (VF) loss in glaucoma is typically measured using white-on-white perimetry,^2^^,^^4^ where the subject is asked to fixate on a central target while stimuli of varying intensities are projected at various retinal locations. The subject presses a button every time a light stimulus is perceived. This information is then used to compute the retinal sensitivity at each tested location.^5^

In recent years, there has been increasing interest in the study of macular damage in glaucoma.^6^^,^^7^ Macular involvement can seriously impact the visual function and vision-related quality of life of patients and is now recognized to be a feature of glaucoma even in early stages.^8^^–^^11^

The macula can be assessed with high precision with both functional and structural tests. For example, exhaustive thickness measurements of the posterior pole can be obtained through high-density OCT scans. Likewise, the 10-2 perimetric grid provides a detailed sensitivity map of the macular region, with an examination resolution of 2°.^12^^–^^15^ These measurements have been combined to study the structure–function relationship in glaucoma.^13^^–^^17^ Such analyses require establishing the spatial correspondence between tested locations and measured thickness values. This is challenging as a consequence of the radial displacement of the retinal ganglion cells in the macula^18^^,^^19^ and the accuracy of spatial mapping of perimetry onto structural maps.^15^ The latter is especially important in the macula, as inaccurate mapping can potentially nullify any advantage offered by the high spatial resolution of the measurements. We explore this challenge in this study.

Any mapping scheme is based on certain assumptions. Usually, it is assumed that the center of fixation, or preferred retinal locus (PRL), coincides with the anatomical fovea; however, patients can exhibit eccentric fixation, especially with advanced macular damage,^20^^,^^21^ even in glaucoma.^15^^,^^22^ Moreover, some researchers have proposed that the 10-2 VF grid should be rotated to match the anatomical fovea–disc axis.^13^^,^^16^ Such an assumption is not supported by evidence on how stimuli are projected during perimetry. Another major hurdle is fixation instability. In fact, subjects might not be able to maintain steady fixation on the central target throughout the VF test.^23^^–^^25^ This can result in projection of stimuli on the retina away from the intended location. In an attempt to solve this issue, fundus perimetry has been introduced.^21^^,^^24^^,^^26^^–^^29^ Fundus perimetry employs tracking of eye movements through continuous retinal imaging and actively compensating for eye movements when projecting the stimuli. Originally designed to test patients with age-related macular degeneration,^21^ fundus perimetry has been successfully employed in glaucoma to improve test–retest variability and structure–function relationships.^17^^,^^26^^–^^28^ Importantly, fundus perimetry locks the stimulus location on a reference image of the subject's retina, providing precise landmarks to accurately link perimetric data to OCT maps.^15^ Finally, as a useful byproduct of the tracking procedure, detailed two-dimensional information on the fixation behavior of the subject during the test is provided.^21^^–^^25^^,^^30^^,^^31^

In this work, we combine structural information from an OCT device and functional data collected with a fundus perimeter from healthy subjects and glaucoma patients. The objective was to use projection and fixation data from fundus perimetry to (1) establish whether grid rotation along the fovea–disc axis as a preferred mapping scheme is supported by evidence, and (2) quantify the spatial error of stimulus projection in perimetry when eye movements are not compensated for, as in traditional VF testing.

## Methods

### Data Collection

This was a retrospective analysis of data collected for a previously published study.^15^ The study adhered to the tenets of the Declaration of Helsinki and was approved by the local ethical committee (Humanitas–Gavazzeni Hospital Ethical Committee, reference number 161/18gav).^15^ After obtaining written consent, we collected data from 17 visually healthy subjects and 31 glaucoma patients. All glaucoma patients and nine of the healthy subjects had previous experience with perimetry, but not with the fundus perimeter used in this study. All subjects were instructed to maintain central fixation, as in traditional perimetry. The data collection has been described elsewhere.^32^ In brief, spectral-domain OCT high-density volume scans (121 vertical b-scans) of the macular region were acquired with a fundus tracking device, the Spectralis (Heidelberg Engineering, Heidelberg, Germany). Axial length was measured with an IOLMaster V3 A-scan (Zeiss Meditec, Dublin, CA). The 10-2 VF test was performed on these 38 subjects with a Compass (CMP) fundus perimeter (CenterVue, Padua, Italy). Twenty additional glaucoma patients were also tested with a custom small grid for the main experiment and were not included in this analysis.^32^ The CMP has a tracking speed of 25 Hz using an infrared fundus camera, with an approximate resolution of 32 pixel/deg. The theoretical maximum resolution of the tracking is equivalent to that of the camera (0.03°) but can be reduced by blurred or low-quality images. The device has a background illumination of 31.5 apostilb (asb) and uses a Bayesian testing strategy (Zippy Estimation Through Sequential Testing, or ZEST)^28^^,^^33^ to determine retinal sensitivity. The device tracks the eye for 10 seconds at the beginning of the test to determine the PRL on the retina.^23^ The testing grid is then centered on this location, which might be different from the anatomical fovea. The position of the tested locations is calculated in degrees from fixation (PRL) as in conventional perimetry.

### Analysis of Fixation and Projection Data

We extracted the complete tracking recordings of fixation during the test for each exam; these are composed of retina displacements over time (in milliseconds) in the horizontal and vertical direction (in degrees) with respect to a reference image acquired at the beginning of the test.^15^^,^^23^ We also extracted the time, intensity, position relative to the PRL, and response time (button press) of all the stimulus projections occurring during the test.^15^ The two tracks (fixation and projections) were then matched using the time reference to quantify fixation behavior before and after each stimulus projection.

First, we used this information to detect eye movements that were likely caused by gaze attraction from seen stimuli. We called these movements evoked displacements. The methodology for this analysis has been presented previously (Modarelli A, et al. *IOVS*. 2018;59:ARVO E-Abstract 5131) and is reported in detail in the Appendix. In brief, a filter identifies eye movements, above an individualized noise threshold, directed toward a stimulus projection. Either these eye movements can be removed from the fixation track, to give a more robust quantification of fixation, or they can be analyzed as a separate component of spatial projection error (see below).

To quantify fixation behavior, we calculated the 95% bivariate contour ellipse area (95% BCEA)^23^^,^^25^ of fixation positions before and after removal of the evoked displacements. We also calculated the average displacement of fixation from the PRL during the test, as this can be easily related to common fixation tracks provided by traditional perimeters,^34^^,^^35^ such as the Humphrey Field Analyzer (Zeiss Meditec).

### Structural Mapping

Fundus images from the CMP and the Spectralis can be used to match VF test locations to structural maps.^15^ For this analysis, we were only interested in detecting the anatomical fovea and the position of the ONH. The former was automatically detected using a template matching technique on the OCT measurement of the whole retinal thickness,^15^ and the latter was manually identified on the wide-field CMP image.

When a geometric projective transformation^15^ has been estimated by matching the fundus images from the two devices, the positions of the anatomical landmarks, the tested locations, and the fixation track can be mapped into the coordinates of either device.

**Figure 1.**
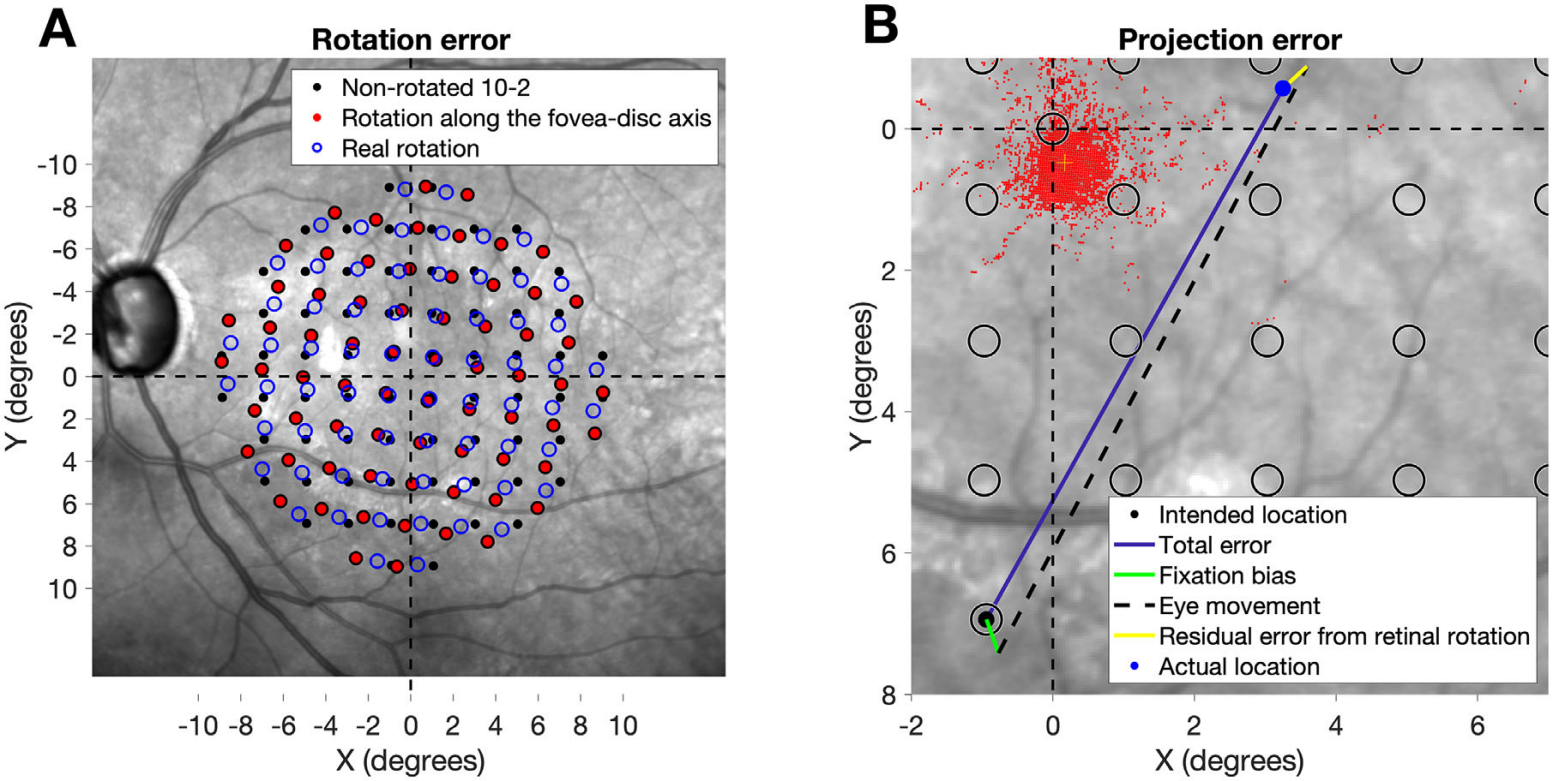
(**A**) Spectralis fundus picture showing different mapping schemes. All are centered in the fovea. The *filled black points* represent the non-rotated 10-2 grid (i.e., assuming the horizontal axis of the VF is horizontal on the retina). The *filled red points* show the 10-2 grid rotated to match the horizontal axis with fovea–disc axis of the subject. Finally, the *empty blue circles* represent the grid with the real observed rotation from the CMP. The (0,0) coordinate represent the location of the anatomical fovea. (B) Calculation of the projection error. The different segments show different component of the error. The *empty black circles* represent the intended test locations for the 10-2 grid referenced to the anatomical fovea. The *small red dots* represent the cloud of fixation positions during the exam. The offset of its center from the anatomical fovea indicates the fixation bias. The (0,0) coordinate represents the location of the anatomical fovea.

In this study, we assumed that the retinal rotation during the VF test was the one observed in the fundus image from the CMP; therefore, when stimulus locations were reported on the maps from the Spectralis, such a rotation was preserved. It is important to note that the projection of the 10-2 grid in the CMP is analogous to any non-fundus tracked perimeter; that is, stimuli are presented at a predefined eccentricity with no rotation, regardless of the relative position of the fovea or ONH. However, in contrast to other devices, the retinal image can be used to assess the rotation of the eye relative to the grid during the test. The results from this analysis are therefore generalizable to structure–function analyses performed with any perimeter. For objective 1, the effect of artificial grid rotation (to align the horizontal axis of the VF grid to the fovea–disc axis) on the mapping error was calculated as the Euclidean distance between the locations of the rotated or non-rotated grids, centered on the fovea, and the actual locations on the Spectralis maps (Fig. 1A). This approach preserves the real observed retinal rotation but removes fixation bias (see next section); therefore, all grids were assumed to be centered in the fovea, and the effect of rotation was isolated.

### Quantification of Projection Errors

Our main goal was to quantify projection errors occurring when eye movements are not compensated for. This happens in conventional perimetry when performing structure–function analysis. We defined projection errors as the spatial distance (in degrees) between the actual location of the projection on the retina (had there been no fundus tracking) and its intended location, in this case the stimulus coordinates of a 10-2 grid centered on the anatomical fovea. One important aspect of fundus perimetry is that it locks the stimuli on fixed positions on the retina based on the initial estimation of the PRL; however, this is not what happens in conventional perimetry. Therefore, in our calculations for objective 2, we estimated the actual projection location during the test by adding the last fixation offset (recorded immediately before the stimulus projection) to its intended position, in degrees from the anatomical fovea. Finally, the small differences in retinal rotation between the fundus images from the CMP and the Spectralis (Fig. 1B) were also added.

We considered the total projection error for each test to be composed of two different additive elements:

1. *Fixation bias*—This is a consistent offset of the PRL from the anatomical fovea. In our analysis, the fixation bias was calculated as the average offset in the horizontal and vertical directions of the fixation positions after the evoked displacements had been removed. The fixation bias was then removed before calculating the following components.
2. *Eye movements*—The gaze displacements that occur during the test can be classified as follows:
• *Evoked displacements*—These are eye movements caused by gaze attraction from perceived stimuli. The exact method for their detection is explained in the Appendix. To quantify their effect, the error for each projection was identified as being a consequence of an evoked displacement if such a displacement happened during the previous presentation. Therefore, an evoked displacement caused by one stimulus presentation is assumed to influence the error of the following projection. This happens if the subject does not return to central fixation after the evoked displacement.
• *Random displacements*—These are eye movements caused by random fixation instability. They are composed of all the calculated projection errors that are not attributed to evoked displacements.

We finally defined the unbiased error as the ensemble of evoked and random displacements (i.e., after removing the fixation bias from all projection errors). All calculations for the image and track analyses were performed in MATLAB (The MathWorks, Inc., Natick, MA).

### Statistical Analysis

Changes in the 95% BCEA before and after removal of evoked displacements^23^^,^^25^ were modeled using a generalized linear model (GLM) with a gamma distribution of the statistical error and a log link function for the BCEA. Such an approach accounts for the skewed distribution of the BCEA (strictly positive) and models the effect of the predictors as proportional (additive in log-scale). This is consistent with previous reports studying the log-transformed BCEA. Differently from a log transformation, GLMs allow a direct estimate of the mean and standard error in the original scale of the dependent variable. Random intercepts were added to account for the repeated measures from the same eye (BCEA with and without evoked displacements). The marginal (population) estimates from a mixed model with a nonlinear link function (log, in this case) are, however, conditional to specific values of the random intercept.^36^ Unconditional marginal estimates were derived numerically using the glmmadaptive package^37^ for R (R Foundation for Statistical Computing, Vienna, Austria). Differences in 95% BCEA with and without evoked displacements between the healthy and glaucoma cohort were calculated through a single model that included an interaction between the group (healthy or glaucoma) and the type of displacement (random or evoked).

The projection error in our analysis is defined as a distance; the distribution of this variable is also expected to be positive and right-skewed; therefore, GLMs with a proportional effect of the predictors can be suitable in this case, as well. However, for ease of interpretation, it is convenient to instead model the effects on the error as additive. Hence, we used simple linear mixed-effect models, with a random intercept term to account for correlations among observations from the same test, for all the statistics describing the projection errors (lme4 package for R).^38^ The effect of evoked displacements was coded for each presentation as a binary fixed-effect predictor in the mixed model. The specific effect of evoked displacements was analyzed using the unbiased error. The linear model expressed the difference in projection error for presentations following likely evoked displacements compared to the other presentations. The differences in the frequency of evoked displacements between the healthy and glaucoma cohort were studied using a logistic regression with random intercepts. This is also a GLM with a nonlinear link function (logit) and random effects. The population estimates were therefore also obtained with the gmmadaptive package.^37^ Differences between random and evoked displacements between the healthy and glaucoma cohort were calculated through a single model that included an interaction between the group (healthy or glaucoma) and the type of displacement (random or evoked).

Age (years) was always included as a covariate, except when calculating the error introduced by rotation, as this was not dependent on functional factors. Age-adjusted estimates and 95% confidence intervals (CIs) are reported for the average age of the overall sample (61 years). The level of statistical significance for the analyses was set to 0.05. The Tukey–Kramer method was used to correct for multiple testing when performing pairwise comparisons. All statistical analyses were performed in R

## Results

Demographic characteristics of the final sample are reported in Table 1. On average, the healthy cohort was younger than the glaucoma cohort. The two cohorts overlapped in the range between 34 and 62 years of age, which included 20 subjects (43%). The fixation track could not be extracted for three healthy subjects. One glaucoma subject was excluded because, despite correct initial alignment and PRL detection, the center of fixation was several degrees away from the central target throughout the exam. The patient reported seeing a ghost image of the central target projected superiorly.

**Table 1.** Demographics of the Sample

	Median (95% Quantile)	
	Healthy (*n* = 17)	Glaucoma (*n* = 30)
Age (y)	42 (27, 60)	74 (44, 87)
Axial length (mm)	24.17 (22.06, 25.84)	24.18 (22.24, 26.33)
BCVA (dB)	1.0 (0.80, 1.00)	0.70 (0.22, 1.00)
HFA 24-2 (dB)	—	–14.42 (–27.67, –3.12)
CMP 10-2 MD (dB)	–0.28 (–1.89, 0.56)	–13.00 (–26.16, –6.40)
Exam duration (min)	6.6 (5.7, 14.5)	9.9 (7.3, 16.5)

The 24-2 data for glaucoma patients were obtained from clinical charts. BCVA, best-corrected visual acuity; HFA, Humphrey Field Analyzer.

### Mapping Error Introduced by Grid Rotation

**Figure 2.**
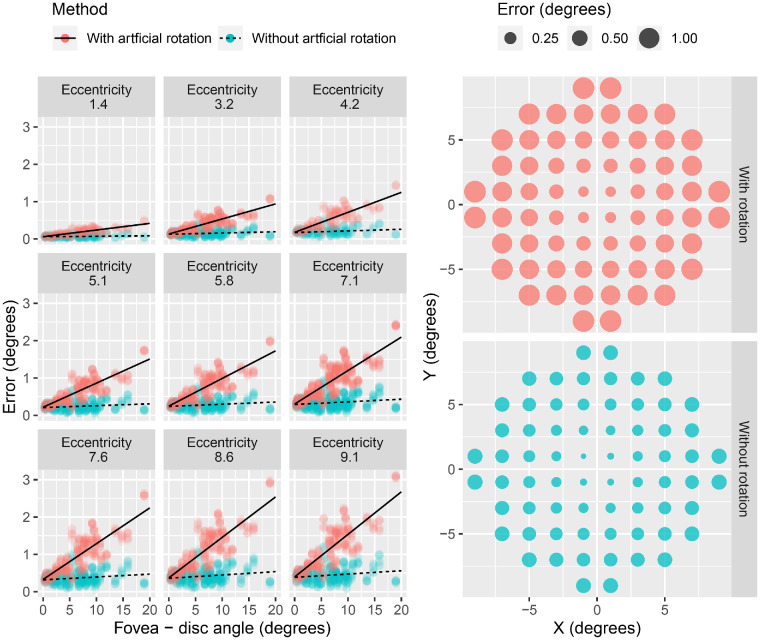
The left panel shows the systematic error introduced by artificial grid rotations at different eccentricities according to the measured fovea–disc angle. The right panel shows the mean error estimated from the model at different locations with and without grid rotation to match the fovea–disc axis.

The effect of aligning the horizontal axis of the 10-2 grid with the fovea–disc axis (rotation) is shown in Figure 2. The reference was the actual rotation of the grid observed with the CMP. The mean error for the rotated grid was 0.80° (95% CI, 0.73°–0.86°), and for the non-rotated grid it was 0.30° (95% CI, 0.23°–0.36°) (*P* < 0.001). Grid rotation introduced a systematic error that was larger for more eccentric locations and increased proportionally with the amplitude of the fovea–disc angle (*P* < 0.001) (i.e., with the amount of rotation required) (Fig. 2). No significant systematic error was introduced with the non-rotated grid.

### Projection Errors Due to Fixation Movement

**Figure 3.**
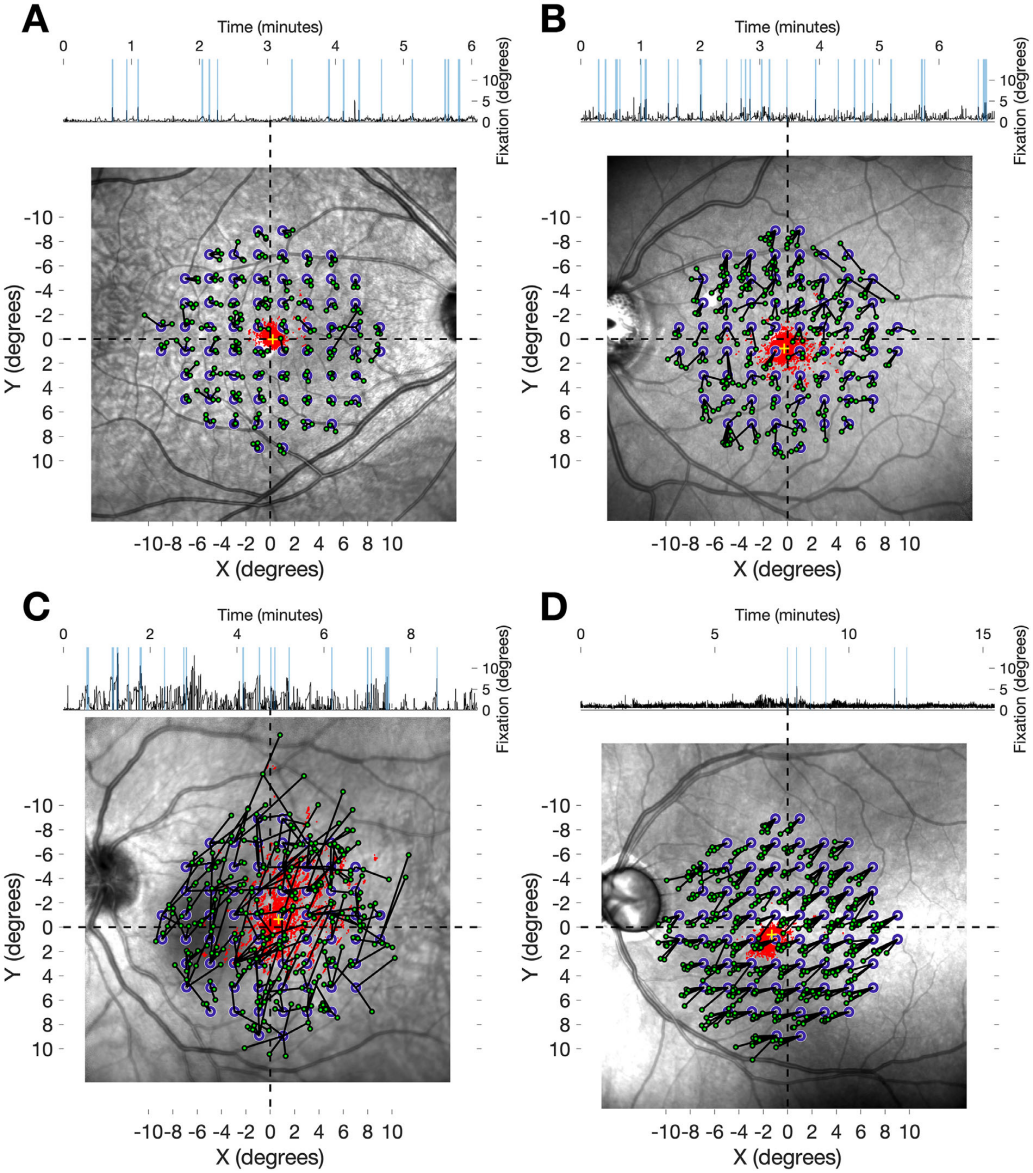
Examples from four different subjects of projection errors during a 10-2 VF test. All images are centered on the anatomical fovea. The *small red dots* represent the cloud of fixation positions during the test. The *yellow cross* corresponds to the fixation bias. The *empty* *blue* *circles* represent the intended position of the tested location. The *small* *green* *circles* represent the actual location of each projection on the retina, connected to its intended location by a *black line*. The top track represents the fixation displacement from the initial PRL. The shaded *blue vertical bands* in the track indicate evoked displacements. (A) Small fixation bias, stable fixation; (B) larger fixation bias, more unstable fixation; (C) extremely chaotic fixation; (D) stable fixation with large fixation bias.

Projection errors from four different example subjects are reported in Figure 3.

Age-corrected estimates of the 95% BCEA were lower in glaucoma patients than in healthy subjects, but the difference did not reach significance (*P* = 0.062) (Table 2). The 95% BCEA recalculated excluding the evoked displacements was significantly smaller, both in glaucoma patients and healthy subjects (*P* < 0.001) but showed no significant differences between the two groups (*P* = 0.886). Healthy subjects showed a significantly larger reduction in 95% BCEA when evoked displacements were removed compared to glaucoma patients (*P* = 0.034). The age-corrected 95% BCEA was also significantly positively correlated with the 10-2 mean deviation (MD) in glaucoma subjects (4.9% increase/dB; *P* = 0.014), but no significant relationship could be found between the MD and the 95% BCEA after the removal of evoked displacements (*P* = 0.265). The frequency of evoked displacements was significantly higher (*P* = 0.047, logistic regression) in healthy subjects (6%; 95% CI, 4%–8%) than in glaucoma patients (4%; 95% CI, 3%–5%).

**Table 2.** Fixation Metrics and Projection Errors

	Median (95% Quantiles)		Age-Corrected Estimates of the Mean (95% CIs)	
	Healthy	Glaucoma	Healthy	Glaucoma
Fixation				
95% BCEA (deg^2^)	3.91 (0.38, 21.45)	3.76 (0.64, 20.98)	8.02 (4.78, 13.46)	4.09 (2.93, 5.7)
95% BCEA (deg^2^) without evoked displacements	0.68 (0.23, 3.45)	2.54 (0.34, 6.31)1.98 (1.05, 3.75)	1.86 (1.21, 2.86)	
Fixation bias (deg)	0.47 (0.06, 0.95)	0.52 (0.18, 1.55)	—	—
Projection errors (deg)				
Total	0.63 (0.16, 2.27)	0.73 (0.19, 2.45)	0.99 (0.75, 1.23)	0.89 (0.73, 1.05)
Unbiased				
Evoked	0.53 (0.13, 3.13)	0.49 (0.12, 2.88)	1.04 (0.82, 1.25)	0.81 (0.67, 0.96)
Random	0.43 (0.11, 1.73)	0.46 (0.11, 1.68)	0.78 (0.56, 0.97)	0.55 (0.41, 0.68)
Time interval between presentations (s)	1.43 (0.89, 2.32)	1.7 (1.1, 2.56)	1.54 (1.46, 1.63)	1.76 (1.68, 1.84)

The mean values are estimated at the overall average age of the sample (61 years). The estimates for evoked and random errors quantify the amount of unbiased error for presentations following likely evoked displacements (evoked) and all the other presentations (random).

**Figure 4.**
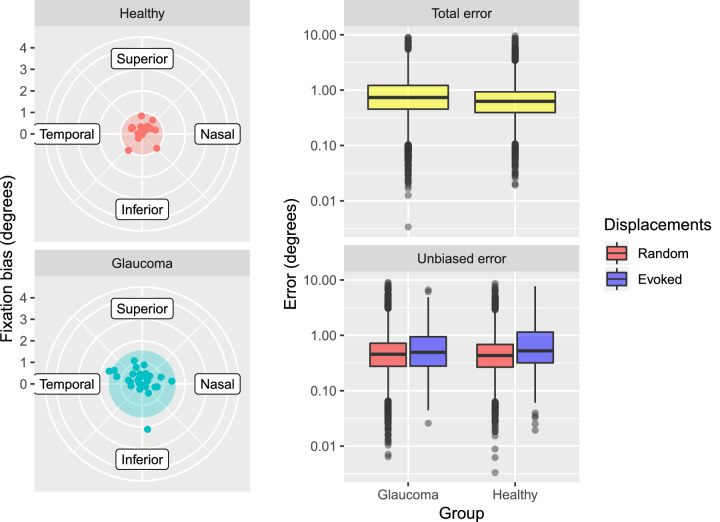
The left panel shows the fixation bias of each subject. The center of the polar plots represents the anatomical fovea. The dots represent the position of the average fixation during the test. The shaded circle encloses the 95% quantile value of the distance of the center of fixation from the fovea for each group. The panel on the right shows the total error (*top*) for glaucoma and healthy subjects and the unbiased error (*bottom*) broken down into evoked and random displacements. The spacing of the vertical axis is in log_10_ steps.

Average fixation bias was greater (Table 2; Fig. 4, left panel) for glaucoma patients but this difference did not reach statistical significance (*P* = 0.15). Age-corrected estimates for mean projection error were not significantly different between glaucoma patients and healthy subjects (Table 2; Fig. 4, right panel), neither for the total error (*P* = 0.53) nor for the unbiased error (*P* = 0.13). Removing the fixation bias significantly reduced the error in both healthy and glaucoma subjects (*P* < 0.001), with a significantly larger effect on glaucoma patients (*P* < 0.001). Evoked displacements significantly increased the error in both glaucoma patients (*P* < 0.001) and healthy subjects (*P* < 0.001), and the effect was not different between the two groups (*P* = 0.839).

**Figure 5.**
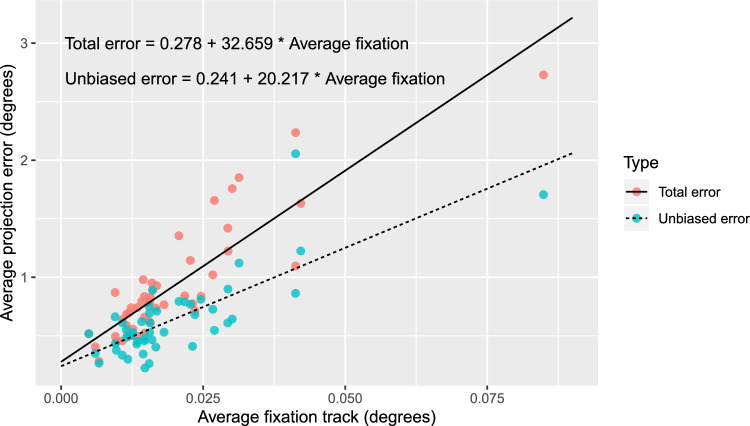
Linear regression of the average total and unbiased error according to the average displacement of the fixation track for each subject. Equations of the linear fit are given.

Both the total and the unbiased average errors were very well predicted by the average fixation track displacement (*R*^2^ = 0.77 for the total error; *R*^2^ = 0.60 for the unbiased error; *P* < 0.001) through a simple linear relationship (Fig. 5).

## Discussion

### Mapping Error Introduced by Grid Rotation

The first objective of our work was to test whether rotating the 10-2 grid to match the fovea–disc axis was the best mapping scheme for structure–function analysis. We compared the error with and without rotation using the actual eye rotation observed with a fundus perimeter as a ground truth. With our data, we did not find any evidence to support that grid rotation provides a better mapping of the tested locations on the retina. Moreover, we found that rotating the 10-2 grid introduced a systematic error proportional to the fovea–disc angle. This finding has some important consequences for previously published results,^13^^,^^16^ where grid rotation was applied. Because most of these results relied on pointwise topographical analyses, their validity now seems questionable. These studies did not use a fundus perimeter, so the actual location of the stimuli cannot be known; however, our findings easily generalize to conventional perimetry because the head positioning of the patient and the projection of the stimuli are identical.^28^ The CMP, in fact, projects the 10-2 exactly as a traditional perimeter would, with no regard for the relative position of fovea and the ONH. Of course, with the aid of imaging and fundus perimetry, the 10-2 grid could be forcibly aligned with the fovea–disc axis. However, further studies, such as on how the anatomy of retinal ganglion cells changes with the position of the ONH, are necessary to understand whether such a change would provide any advantage in structure–function analyses and diagnostic ability.

### Projection Errors Due to Fixation Movement

The second objective was to quantify how eye movements contributed to errors in the projection of perimetric stimuli on the retina. Here, we used fixation and projection data from the CMP and structural data from a spectral-domain OCT to model what would happen in conventional perimetry. We specifically isolated the effect of gaze attraction from projected stimuli in what we called evoked displacements. We found that removing these evoked displacements from fixation data significantly shrank the 95% BCEA in both healthy subjects and glaucoma patients (*P* < 0.001). This reduction was significantly more pronounced in healthy subjects (*P* = 0.034), as evoked displacements were significantly more frequent in this group (*P* = 0.047). This could be partially explained by the fact that healthy subjects were less experienced with perimetry than glaucoma patients; however, rather than an actual change in fixation behavior, we attribute this difference to a higher number of seen presentations in healthy subjects, resulting from the way threshold strategies probe VF sensitivity.^5^^,^^33^ This is also corroborated by the finding that the 10-2 MD was positively correlated with the 95% BCEA in glaucoma subjects (larger for more initial damage), but such a relationship was not significant when evoked displacements were removed. Notably, all glaucoma subjects were experienced test takers. We then found that the projection error of stimulus presentations preceded by an evoked displacement was significantly increased compared to the rest of the presentations (*P* < 0.001) (Fig. 4). This is not an obvious result, as the time interval between presentations (Table 2) could allow subjects to return to central fixation. Previous work investigating fixation area in fundus perimetry found a significantly increased 95% BCEA in glaucoma patients.^30^^,^^31^ In a previous study,^23^ however, we analyzed data from the PRL assessment phase in the CMP on a different dataset and found no difference between healthy subjects and glaucoma patients, irrespective of their level of damage, although there was a significant difference in other fixation metrics.^23^ This is confirmed by the results of this study, as no difference was found in the 95% BCEA between glaucoma and healthy subjects. Interestingly, Longhin et al.^30^ reported an increase in BCEA during the perimetric test compared to the initial PRL assessment phase, during which time no stimuli were projected. They speculated that this spread in fixation area could be the effect of projected stimuli attracting fixation, and this is consistent with our findings.

Another component of the error that we analyzed was the fixation bias. We could not find a statistically significant difference between healthy subjects and patients with glaucoma (*P* = 0.12). Yet, removing the fixation bias significantly reduced the projection error in both groups, with a significantly larger effect in glaucoma patients (*P* < 0.001). This apparent discrepancy can be explained by the sample size; in the first analysis it was limited to the number of subjects included in the study (*N* = 47), but the second result is based on the analysis of each presentation from the VF tests on those subjects (*N* = 14,343 data points). This finding has important consequences. Regarding structure–function analyses, it obviously challenges the notion that the center of the perimetric grid should be placed in the fovea when mapping perimetric thresholds onto structural data. Similar results have been shown for patients with other optic neuropathies with central damage.^39^ This issue can be addressed by fundus perimetry, as the position of the stimulus projection is known with higher precision and can be used to obtain more accurate mapping.^15^^,^^22^ Other solutions might include methods based on structural analyses of the macular damage or on ad hoc fixation analyses derived from other fundus tracking devices, such as the Spectralis.^39^ Additionally, such a consistent shift in fixation has important consequences for deriving normative databases in perimetry. At present, the additional variability introduced by a fixation bias, which effectively changes the location of the projected stimuli, is not taken into account. It has to be noted that this latter issue is not solved by fundus tracked perimetry, as the center of the perimetric grid is determined by an initial functional assessment of the PRL. One possible solution would be to integrate fundus perimetry and OCT imaging to detect the location of the anatomical fovea and ensure that this is used as the center of the perimetric grid instead.

We also showed that the error can be reliably predicted from the fixation track (Fig. 5). This could be useful for researchers not using fundus perimetry to determine the amount of error in their measurements. In fact, the fixation tracks produced by fundus perimetry can be easily related to similar graphs produced by traditional perimeters with pupillary fixation monitors. A method for the quantitative analysis of these tracks has been proposed by Ishiyama et al.,^34^^,^^35^ for example; however, studies are needed to establish the exact correspondence between the results of fundus and pupillary tracking.

Finally, it is important to notice that our work did not aim at quantifying the effect of eye movements on perimetric sensitivity. We instead estimated the error induced by fixation and artificial grid rotation when reporting retinal sensitivities onto structural maps. Previous work thoroughly investigated the effect of eye movements on perimetric sensitivity.^40^^–^^42^ Also, in our previous report on the CMP,^28^ we showed that, despite improving test–retest variability for global indices, fundus tracking had only a modest effect on discrimination ability compared to traditional perimetry. However, our previous study compared two different devices, with two different testing strategies, using a 24-2 grid, whose locations were 6° apart.^28^ This could have limited detecting the impact of tracking. In fact, even with very chaotic fixation (Fig. 3C), errors ≥ 6° are extremely unlikely (0.2% in our sample, compared to 3.8% ≥ 2°) (see Supplementary Fig. S1). A more precise quantification of the effect of fundus tracking on perimetric sensitivity and test–retest variability using a 10-2 grid will be the objective of future work.

### Limitations

One limitation of our work is the relatively small sample size; however, we exploited the large amount of information contained in each VF test by analyzing each projection. As for many other fundus tracking devices, the CMP fundus tracking speed of only 25 Hz is a technical limitation for our study. Therefore, we were only able to analyze fixation up to this resolution, and faster eye movements are likely to have gone undetected. Faster tracking is available with pupillary monitors^43^; however, these have the disadvantage of not using retinal images as a reference, eliminating an essential piece of information for our analyses. Nevertheless, further studies using pupillary tracking would be extremely useful to better characterize the effect of evoked displacements both on projection accuracy and on fixation metrics, such as the BCEA.

Finally, the structural and functional tests were not performed through the same optical system but instead relied on a post hoc matching of fundus images from two devices. This could induce further uncertainty and could only be solved with an integrated OCT–fundus perimeter system.^44^

## Supplementary Material

Supplement 1

## Figures and Tables

**Figure A1.**
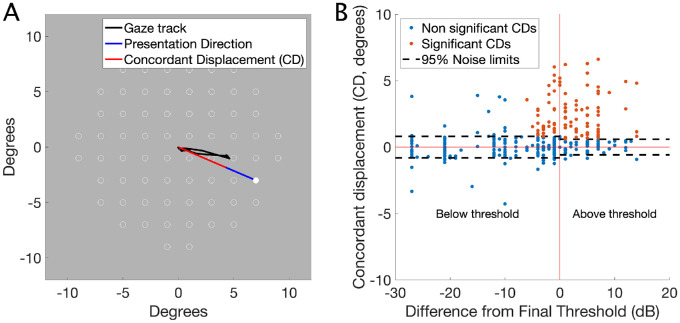
The *red track* in the top image represents the gaze displacement evoked by the projection of the stimulus, indicated by the *solid white dot*, overlaid on the fundus image. The bottom graph shows the same displacement (in *blue*) in visual field coordinates. The *black segment* represents the PRL–stimulus direction, whereas the *red segment* represents the orthogonal projection of the maximum displacement on the *black segment* (the concordant displacement, CD). (B) *Red dots* represent the significant positive CDs (evoked by the stimulus). Dashed lines represent the 95% noise limits calculated from the negative CDs and reflected on the positive upper half of the graph. Different noise levels were calculated for projections below (negative on the horizontal axis) and above (negative on the horizontal axis) the threshold.

## Appendix. Identification of Evoked Displacements

For each exam, the fixation track was matched with the sequence of the presentations, which reported the intensity, location, and time of the stimulus projections. The whole fixation track was subdivided into segments delimited by projections times. For example, the fixation segment between the onset of one stimulus (*t*_0_) and the following projection (*t*_1_) was assigned to the projection started at *t*_0_. Fundus tracking ensured that the stimuli were projected at the intended retinal location (relative to the PRL). Therefore, for each segment, the coordinates of the first position were subtracted from the rest of the segment positions, and all of the following positions within a segment were referenced to the last tracked position before the presentation of the stimulus, set as zero. For each presentation, the displacement was calculated as the maximum distance from zero reached within the fixation segment (Fig. A1A). To quantify how much each displacement was directed toward the stimulus, the orthogonal projection of the displacement onto a line joining the center of the grid (the initial PRL) with the location of the stimulus was calculated (Fig. A1A). We named this the concordant displacement (CD). So, a displacement exactly reaching the stimulus location would produce the maximum CD, whereas displacements at 90° with respect to the stimulus direction would produce a null CD. Finally, a displacement pointing in the opposite direction would produce a negative CD. These values can then be used to build a graph where the vertical axis reports the CD (in degrees) and the horizontal axis reports the intensity of the stimulus projection at each location as a difference from the final threshold determined for that same location (in dB, Fig. A1B). Therefore, negative values in the horizontal axis indicate stimulus intensities dimmer than threshold, and positive values indicate intensities brighter than threshold.

To detect significantly positive CDs, indicating evoked displacements, we estimated the individualized noise from the negative part of the graph on the vertical axis, under the assumption that negative CDs were just a consequence of random gaze movements during the projection of the stimuli. Hence, we calculated a 5% noise threshold that was then reflected on the positive part of the graph. We considered as evoked displacements all the segments with a CD value above the noise threshold and evoked by a projection within at least 10 dB below threshold (–10 dB on the graph, Fig. A1B). It is important to notice that this filter does not simply remove large gaze movements based on their magnitude but acts only on those that are likely caused by stimulus projections.
