# Supplementary material for: Systematic and Random Mapping Errors in Structure – Function Analysis of the Macula
Source: Transl Vis Sci Technol. 2021 Feb 16;10(2):21. doi: 10.1167/tvst.10.2.21 (PMC7900880; doi:10.1167/tvst.10.2.21)
Supplement: Supplement 1 [file tvst-10-2-21_s001.pdf]

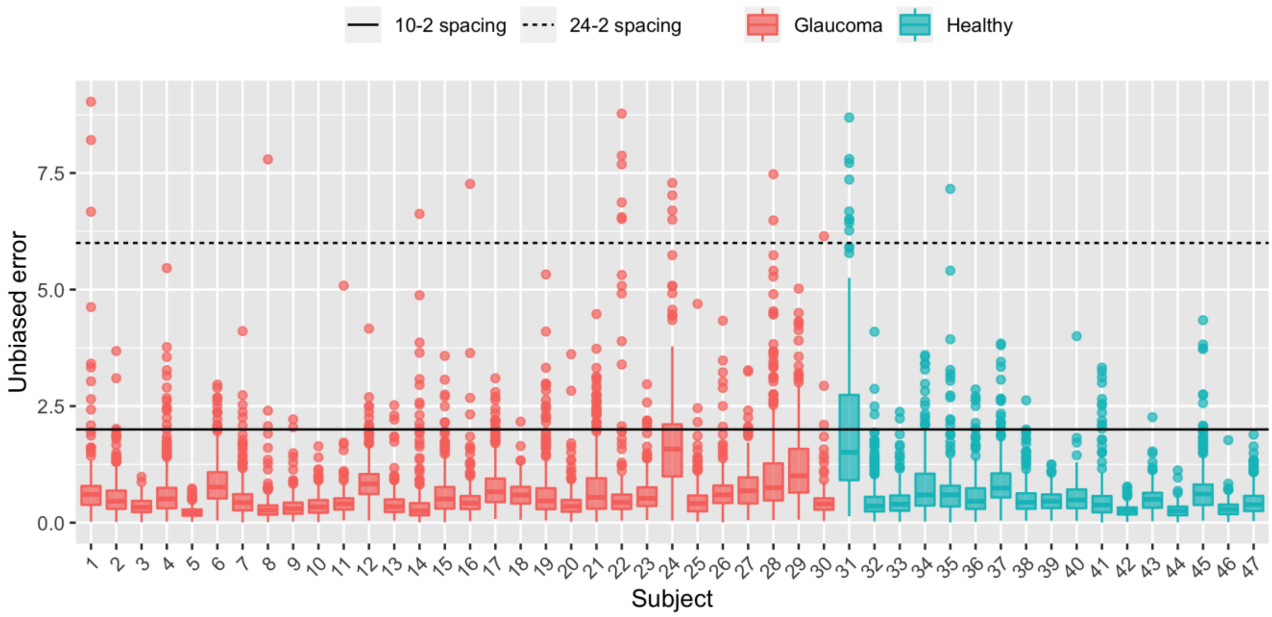

Supplementary Figure. Box-plots of the unbiased error for each subject. The boxes include the interquartile range, the whiskers extend to the 95% quantiles and the horizontal midline represents the median. The two horizontal black lines represent the spacing between locations of a 10-2 grid and of a 24-2 grid as a reference.
